# Vibrational Biospectroscopy: An Alternative Approach to Endometrial Cancer Diagnosis and Screening

**DOI:** 10.3390/ijms23094859

**Published:** 2022-04-27

**Authors:** Roberta Schiemer, David Furniss, Sendy Phang, Angela B. Seddon, William Atiomo, Ketankumar B. Gajjar

**Affiliations:** 1 Division of Child Health, Obstetrics and Gynaecology, University of Nottingham, Nottingham NG5 1PB, UK; ketankumar.gajjar@nuh.nhs.uk; 2 Mid-Infrared Photonics Group, George Green Institute for Electromagnetics Research, Faculty of Engineering, University of Nottingham, Nottingham NG7 2RD, UK; david.furniss@nottingham.ac.uk (D.F.); sendy.phang@nottingham.ac.uk (S.P.); angela.seddon@nottingham.ac.uk (A.B.S.); 3 College of Medicine, Mohammed Bin Rashid University of Medicine and Health Sciences (MBRU), Dubai P.O. Box 505055, United Arab Emirates; william.atiomo@mbru.ac.ae

**Keywords:** endometrial cancer, uterine neoplasm, cancer of the endometrium, spectroscopy, Raman spectroscopy, Fourier transform infrared spectroscopy, diagnosis, screening

## Abstract

Endometrial cancer (EC) is the sixth most common cancer and the fourth leading cause of death among women worldwide. Early detection and treatment are associated with a favourable prognosis and reduction in mortality. Unlike other common cancers, however, screening strategies lack the required sensitivity, specificity and accuracy to be successfully implemented in clinical practice and current diagnostic approaches are invasive, costly and time consuming. Such limitations highlight the unmet need to develop diagnostic and screening alternatives for EC, which should be accurate, rapid, minimally invasive and cost-effective. Vibrational spectroscopic techniques, Mid-Infrared Absorption Spectroscopy and Raman, exploit the atomic vibrational absorption induced by interaction of light and a biological sample, to generate a unique spectral response: a “biochemical fingerprint”. These are non-destructive techniques and, combined with multivariate statistical analysis, have been shown over the last decade to provide discrimination between cancerous and healthy samples, demonstrating a promising role in both cancer screening and diagnosis. The aim of this review is to collate available evidence, in order to provide insight into the present status of the application of vibrational biospectroscopy in endometrial cancer diagnosis and screening, and to assess future prospects.

## 1. Introduction

Endometrial cancer (EC) is the sixth most common cancer in women worldwide, with rising incidence affecting women’s survival and health care resources [1] and global projections of progressively increasing disease burden [2]. EC remains under-researched compared with other malignancies, such as ovarian cancer and breast cancer [3]; however, the last two decades have seen a steady increase in EC research activity [4], alongside the involvement of patients and the public in the identification and planning of research priorities [5].

Among patient-supported priorities for womb cancer, of particular interest are the need for: patient risk stratification, individualised diagnostic pathways in case of abnormal uterine bleeding and the development of minimally invasive approaches to monitor treatment and detect disease recurrence [6]. These highlight the inadequacies and limitations of current diagnostic, screening and monitoring approaches and the unmet need for minimally invasive, objective, rapid and accurate alternatives. 

Increasingly more data are emerging in support of the role of vibrational biospectroscopy techniques as cancer diagnostic and screening tools [7,8], as well as the potential role in the assessment of endometrial pathology [9,10]. It is relevant, therefore, to highlight the status quo of the progress made to date in applying vibrational biospectrscopy techniques to EC.

A large number of reviews has been published examining the oncological applications of biospectroscopy [7,11,12,13,14,15,16,17,18,19,20], but to the authors’ knowledge, this is the first to focus on EC.

This review will first summarise current methods of EC diagnosis and screening, with their advantages and limitations. It will then examine current advances of vibrational spectroscopic techniques in EC, assessing how biospectroscopy methods could shape diagnosis, screening and disease monitoring, their relevance to some of the EC top research priorities, and will discuss current limitations and obstacles to clinical application.

## 2. Endometrial Cancer

EC is the most common cancer in women in the developed world, and the fourth leading cause of death due to gynaecological cancer among women worldwide, accounting for an estimated 382,069 new cases and 89,929 deaths in 2018 [1]. Early diagnosis is usually associated with favourable prognosis, but there are significant disparities between high- and low-income countries, indeed the highest annual increase in mortality is recorded in the southern sub-Saharan African region [2,21]. In England, the 5-year survival rate ranges between over 90% for women diagnosed in stage 1 and just 15% for those in stage 4 between 2013–2017 [22,23]. Most cases of EC occur in post-menopausal women, yet the last few decades have seen an incidence increase across all age groups [24]. This trend, particularly in developed countries, is directly linked to the rising prevalence of obesity, one of the most important risk factors for EC [22,25].

EC is a heterogeneous disease [26]. Historically, it was classified by Bokhman [27] into two main types, that differ in aetiology, as well as in clinico–pathological and epidemiological characteristics: Type I, or endometrioid adenocarcinoma, and Type II, which includes non-endometrioid subtypes such as: serous carcinoma, clear cell carcinoma and carcinosarcoma/malignant mixed Müllerian tumours [26,27,28]. Type I cancers are the most common, particularly in Caucasian populations, accounting for approximately three-quarters of all EC. This form is associated with unopposed oestrogen exposure and often arises from a precursor lesion, known as endometrial hyperplasia.

Type II cancers, by contrast, more prevalent for instance in the African–American population, appear to be independent of hormonal risk and are usually not preceded by endometrial hyperplasia. Type II cancers are considered high grade, and display a more aggressive behaviour, with a high risk of extra-uterine disease at first presentation. The prognosis is poorer compared with Type I cancers, with a tendency to recur even in early-stage disease [29,30,31].

This dichotomous classification, while useful, is limited by the molecular and histological heterogeneity within each group, and the now recognised overlap between some Type I and II cancer behaviour. Advances in endometrial cancer genomic and proteomic characterisation have led to a deeper understanding of the biological, pathological and genomic characteristics of cancer subsets and have now provided the basis for a substantial integration of pathological and molecular classifications [32,33]. Indeed, The Cancer Genome Atlas (TCGA) project reported, in 2013, a comprehensive genetic analysis of the most common histological types of EC, identifying molecular mutations that correlate with their clinical behaviour [34]. This pioneering research provided important prognostic information, particularly for a sub-group of high-grade and high-risk endometrial carcinomas, the *POLE* mutated tumours, that have been found to have an excellent prognosis [35]. Due to the potential impact of molecular classification on cancer treatment, the European Society of Gynaecological Oncology (ESGO), the European Society for Radiotherapy and Oncology (ESTRO), and the European Society of Pathology (ESP) now jointly encourage molecular classification of all EC, particularly the high-grade tumours [36].

The practical integration between pathological and molecular classifications in diagnostic pathways remains, however, challenging: from gene sequencing selection and the risk of identifying unwanted or uninterpretable information, to the additional time required to process results, and costs of equipment and specialised laboratory expertise, which may limit the availability of such an integrated approach, particularly in low-income settings.

An alternative approach to a model based on sequencing a panel of selected genes [33] could be to explore all of the range of biochemical features in a sample simultaneously, with the use of vibrational biospectroscopy techniques.

### 2.1. Current Endometrial Cancer Diagnosis and Screening

Timely investigation of women presenting with symptoms, such as post-menopausal bleeding and persistent menstrual irregularities, allows most cases of EC to be identified in early stage.

Ultrasound imaging, hysteroscopy and endometrial biopsy, together with the histopathological tissue analysis, are the current mainstay of EC diagnosis. In addition, magnetic resonance imaging techniques (MRI) are useful in the assessment of depth of myometrium invasion, cervical stromal involvement and lymph node metastasis [37,38,39,40].

However, unnecessary procedure should be avoided, which may expose patients to complications, generate needless anxiety and take up financial resources. Indeed, hormonal imbalance, coagulopathies, benign endometrial lesions and the use of medications including hormone replacement therapy (HRT) are some of the factors associated with irregular and recurrent vaginal bleeding, which may occur in the absence of EC [41]. Consequently, the main challenge in early cancer diagnosis is the appropriate selection of those patients that require investigations and invasive procedures.

#### 2.1.1. Ultrasound Imaging

Ultrasound imaging is a technique, which uses high frequency sound waves to provide information about tissue and organ characteristics. The procedure can be performed by the transabdominal and transvaginal access routes, does not require bowel preparation, is safe and is, overall, well tolerated by patients [42].

Ultrasonography is, however, highly operator dependent. Furthermore, excess adipose tissue interferes with sound wave signals, affecting image quality [42], thus women with high body habitus are at increased risk of suffering diagnostic delays [43].

Measurements of the endometrial thickness using ultrasound imaging are used as a surrogate marker to check for the presence of intrauterine abnormalities [44]. However, ultrasound imaging alone cannot discriminate whether an increased endometrial thickness is secondary to a benign lesion or to malignant disease [44].

Women with post-menopausal bleeding have a 8–11% risk of EC, which justifies the need for endometrial assessment in these patients [45]. The use of endometrial thickness cut-offs of 4 mm and 5 mm leads to the correct identification of 94.8% and 90.3% of EC cases, respectively [46]. However, the test specificity is poor, leading to a high risk of false positive results and, consequently, many unnecessary invasive investigations and biopsies [46,47].

In pre-menopausal women with abnormal uterine bleeding, the diagnostic role of endometrial thickness is controversial, as there can be overlap between physiological thickening caused by sex hormones and that caused by endometrial disease [48]. While it has been suggested that a thickness of <8 mm should be considered as non-hyperplastic [49], and only 1% of endometrial cancers occurs in women < 40 years of age [47], there is still no consensus on the ideal endometrial thickness cut-off in this group of patients [50], thus an alternative or complementary non-invasive triaging tool would facilitate the clinician’s decision-making on when to refer for further invasive diagnostic procedures.

#### 2.1.2. Hysteroscopy

The direct endoscopic visualisation of the endometrial cavity by hysteroscopy, using visible light at 4 to 5× magnification [51], is an invasive procedure that can be performed in order to evaluate the endometrial cavity, to remove lesions such as polyps or small fibroids and to obtain endometrial biopsies.

Hysteroscopy can be carried out both in the outpatient setting and in theatre, under regional or general anaesthetic [52]. Although, overall, the procedure has been shown to be well tolerated, safe, accurate and acceptable, regardless of the setting in which is performed [53,54,55], some patients do experience significant discomfort during outpatient hysteroscopy [55]. Unfortunately, it is difficult to identify this group of patients preoperatively, and for these women a routine hysteroscopic procedure may turn into a painful and traumatic experience. Furthermore, although uncommon, complications may arise, including bleeding, infection and uterine damage [56], and the failure rate of hysteroscopy, where the instrument cannot be successfully introduced into the uterine cavity, has been estimated at 4.2% [55,57].

Well-conducted systematic reviews and meta-analyses found that hysteroscopy is highly accurate for the diagnosis of EC in women with abnormal uterine bleeding [55,58,59] and it is useful at excluding endometrial disease [58,59], although the diagnostic accuracy for endometrial hyperplasia appears to be more modest [55].

Indeed, in its updated 2018 guidance, the UK National Institute for Health and Care Excellence (NICE) now recommends that hysteroscopy can be offered as a first-line investigation for heavy menstrual bleeding, in preference to pelvic ultrasound, if the woman’s history suggests sub-mucosal fibroids, polyps or endometrial pathology [60].

The inevitable consequence of such a diagnostic strategy, however, is the need for a structural re-organisation of healthcare services, in order to absorb the estimated 10,000 extra procedures that would be performed in England each year [61] and their added financial costs. The availability of adequately sized and equipped facilities and investments in recruitment and training of skilled staff are some of the challenges to overcome, in order to implement the new guidance into clinical practice.

#### 2.1.3. Endometrial Biopsy and Histological Analysis

Histological examination of an endometrial biopsy specimen is the current so-called: “Gold Standard” of EC diagnosis. The sample preparation and analyses, required to allow the visualisation of the internal architecture of cells and tissues and identify cancerous features [62] are, however, time-consuming, and can be subject to human error [63].

The condition or quality of an endometrial biopsy must be “adequate” in order to provide the histological diagnosis, but the lack of standard agreement on quality and quantity assessment criteria [64] leaves the decision regarding sample suitability to individual pathologists. This allows for a high inter-observer variability [65] and a risk of diagnostic delay and potentially detrimental consequences for patients. The reported rate of insufficient quality or quantity of endometrial tissue samples for histological diagnosis in post-menopausal women is 31% (range 7–76%) [66], while in pre-menopausal women it is lower, ranging between 2% and 10% [67]. The reasons for insufficient sampling appear unclear: the experience of the operator has not been confirmed to be a determining factor [68,69] and there is wide variance between insufficient sample rates reported in single versus multicentre studies, suggesting that study design may influence the results [55,68,69,70,71].

Importantly, obtaining an endometrial biopsy is not always a straightforward process. Indeed, endometrial sampling fails in approximately 11% of cases (range 1–53%), mostly as a consequence of cervical stenosis [66]. Factors such as the post-menopause, advanced age and nulliparity also appear to be associated with higher failure rates, likely as a consequence of variation in endometrial thickness and anatomical changes that occur in these patient groups [69].

Outpatient endometrial biopsy has mostly replaced traditional dilatation and curettage under general anaesthetic worldwide [72,73,74], as it has shown comparable performance, while being less invasive and more cost effective [75,76]. The diagnostic accuracy of these biopsies were investigated extensively and a number of meta-analyses were published, reporting on sensitivity and specificity in relation to endometrial cancer, endometrial hyperplasia (with and without atypia) and benign endometrial disease [66,67,74,75,77]. Overall pipelle biopsy, with conventional histopathology, appears to be an effective tool to identify endometrial cancer when adequate samples are obtained; however, the test is not as reliable in the case of endometrial hyperplasia, where a negative result only decreases the hyperplasia risk by 2-fold [77].

The potential failure to diagnose or exclude disease after invasive procedures, such as hysteroscopy and endometrial biopsy, is concerning. Coupled with the highly subjective nature of histopathological assessments, it highlights the need for alternative approaches to complement current practice and provide pathologists with additional support in achieving a more objective tissue evaluation. Furthermore, in the context of EC research priorities [6], current diagnostic modalities, despite their established advantages, are insufficient to fully address the need for patient risk stratification and the demand for minimally invasive, individualised, screening, diagnostic and treatment monitoring pathways.

#### 2.1.4. Screening for Endometrial Cancer

Screening is defined by the World Health Organisation (WHO) as “the presumptive identification of unrecognized disease in an apparently healthy, asymptomatic population by means of tests, examinations or other procedures that can be applied rapidly and easily to the target population” [78]. We suggest that an ideal screening test should be accurate, well-tolerated, associated with minimal morbidity and cost-effective.

Women with known Lynch Syndrome already undergo a multimodal surveillance of the endometrium until hysterectomy is performed, due to their high lifetime risk of developing EC [79].

Unfortunately, there is no EC screening test which is accurate and reliable enough to be implemented for the general asymptomatic population [46,47,48,80,81]. Ultrasound imaging, despite its accessibility, safety and low cost, unfortunately lacks the required sensitivity and specificity, as demonstrated by the nested case-control study [80] within the 2016 United Kingdom Collaborative Trial of Ovarian Cancer Screening (UKCTOCS) [82]. The study showed that if the general UK population were screened using an endometrial thickness cut-off of 5 mm, in order to diagnose 80.5% of cancers, for each endometrial cancer or atypical endometrial hyperplasia (AEH) case detected, 58 healthy women would have to undergo additional unnecessary investigation [80]. It is apparent that the modest test accuracy, potential patient risks and added costs, do not justify the implementation of such a screening strategy. There is, therefore, an unmet need to innovate current diagnostic and screening methods, to tackle the increasing endometrial cancer disease burden and allow early disease detection and timely treatment.

## 3. Aim of this Review

Since the turn of the 21st Century, vibrational biospectroscopy technologies have been widely researched [7,8,12,13,83,84,85] and now show significant promise for clinical application as a new type of stain-free pathology on ex vivo tissue, and also promise for in vivo imaging. In the quest for clinical advance, it is timely to examine the potential added value of vibrational biospectroscopy applications with regards to EC.

Here we will review Mid-Infrared Absorption Spectroscopy and Raman Spectroscopy. We aim to highlight progress to date, with particular emphasis on implications for clinical practice and the relevance to EC diagnosis and screening.

## 4. Biospectroscopy

The interaction between electromagnetic radiation and any particular matter results in the measurable linear and nonlinear physical phenomena of absorption, emission, reflection and scattering of the radiation by the matter; measurement of the radiation after its interaction with matter yields information on the makeup and arrangement of the matter and is known as spectroscopy. The measurement is displayed as a spectrum, which is a graphical representation of energy absorption, emission, reflection or scattering by the material as a function of the incoming radiation photon energy (plotted usually as frequency or wavelength). The application of spectroscopic techniques to biological materials is called biospectroscopy and the name was only coined in the 1960s [86].

Mid-infrared Absorption and Raman scattering spectroscopy, are sister-vibrational absorption techniques, being complementary as they are based on different quantum mechanical rules. They are label-free, non-destructive optical methods with the ability to investigate the vibration and rotation of atoms and molecules in biological materials, induced by irradiation by light.

The vibrational spectra that are generated depend on the specific biochemical structure of the sample tested; they provide information on the whole range of molecules within the sample simultaneously, which can, therefore, be interpreted as a unique “signature” or a “fingerprint” of that sample [12].

The alteration of molecular signatures in a cell or tissue, which has undergone disease transformation, can be objectively detected, gaining vibrational spectroscopic techniques a potential role in cancer diagnosis and screening [14,87,88].

### 4.1. Mid-Infrared Absorption Spectroscopy

Mid-infrared (MIR) light is a radiation region of the electromagnetic spectrum of 3–50 microns wavelength, as defined by ISO 20473:2007 [89]. When biological tissues are exposed to MIR light, part of the photon energy can be resonantly absorbed, inducing vibrations; the quantum mechanical selection rules include that there must be a change in dipole moment during the vibration, hence heteropolar chemical bonds are vibrationally stimulated. The intensity and wavelength of each vibration depend on the nature of the chemical bonds and their specific molecular environment, that is its molecular structure [90]. The fraction of energy absorbed by the sample at different frequencies can be quantitatively measured by means of dispersive infrared (IR) spectroscopes [87].

The technology has been further refined and made faster since the 1970s by the introduction of Fourier transform (Ft) IR spectrometers, in which all broadband spectral information is collected simultaneously, and then many times, in order to average and then maximise the signal-to-noise ratio. The raw data obtained, called an interferogram, is then converted using the Fourier transform mathematical algorithm into wavelength intensity, from which the energy absorbed by the sample can be derived [90].

The majority of work reported to date used the technique of Fourier transform Infrared (FtIR) with Attenuated Total Reflectance (ATR) on excised tissue, or extracted body fluids, which overcomes the need for complex sample preparation [90]. Other image acquisition modes include transmission and transflection; these require the use of suitable substrates (e.g., calcium or barium fluoride slides) and longer machine and sample preparation compared with ATR [17]. Transflection was shown to introduce spectral artefacts and so has lost favour [91,92].

### 4.2. Raman Spectroscopy

Raman spectroscopy relies on the principle of inelastic scattering of photons, also known as Raman scattering, and was first discovered by Raman in 1928 [93]. When a monochromatic light source, such as a visible or near-infrared laser, interacts with a sample, most of the light which scatters off is unchanged in energy. However, a very small number of photons will exchange part of their energy with the molecules of the sample: the chemical bonds of the sample become temporarily excited to a virtual state, then relax to a different vibrational state, while the emitted photons shift to a lower (Stokes) or higher (Anti-Stokes) frequency [94]. The shift in frequency, measured by the Raman spectrometer, is indicative of specific vibrational modes of the sample molecules and, therefore, a unique “fingerprint” spectrum can be inferred [88]. The quantum mechanical selection rules of Raman include that the molecular bond should not undergo a change in dipole during vibration, thus favouring homopolar chemical bonds. Hence, Raman spectroscopy is unaffected by water, and is non-destructive and label-free [95]. These characteristics offer technology a high degree of flexibility, with potential applications to the study of fresh, fixed and live tissues and cells [96]. Spontaneous Raman scattering is a rare phenomenon, with a very low probability of occurrence (~1 in 10^8^) [13]. In order to enhance the Raman-scattering signal level, several variations of Raman spectroscopy have been developed, including resonant Raman (RR), coherent anti-Stokes Raman scattering (CARS) and surface-enhanced Raman scattering (SERS) [14]; these are, however, expensive technologies, with a large footprint. Ultimately, the choice of instrument, desired wavelength and spatial resolution will vary depending on the required application. 

### 4.3. Biospectroscopy for Endometrial Tissue Interrogation

The development of effective diagnostic, screening and treatment strategies for endometrial cancer finds its basis in a deep understanding of tissue physiological and pathological processes. In particular, to be clinically useful, a new diagnostic or screening tool should be able to accurately distinguish healthy patients from those with disease.

ATR-FtIR and Raman spectroscopy were used to categorise disease and identify cancer or intra-epithelial neoplasia in a number of excised tissues, such as prostate [97,98,99,100,101], gastrointestinal tract [102,103,104,105,106], brain [95,107,108], breast [109,110,111,112,113,114,115,116], lung [117,118] and skin [119,120,121]. Gynaecological applications include studies of cervical cytology and histopathology [122,123,124,125,126,127], ovarian cancer [128,129] and vulvar disease [130].

With regards to endometrial tissue, vibrational biospectroscopy was successfully applied in preliminary research to the study of its structural architecture [94,131], as well as the classification of cancerous lesions [10,132,133], specific cancer subtypes [132,133] and the identification of cell phenotypes with different drug sensitivity [134] (see Table 1). There is, however, a paucity of literature, and specifically of large studies, compared with other types of diseases.

#### 4.3.1. Assessment of Endometrial Structure

The endometrium is a highly complex regenerative tissue, with proliferation and shedding cycles strictly regulated by the hormones oestrogen and progesterone. The endometrial architecture and cellular turnover are also influenced by the activity of adult endometrial stem cells [135], whose pathological changes may be involved in uterine carcinogenesis and in the development of conditions such as adenomyosis and endometriosis [135,136]. The study of endometrial tissue structure, including stem cell physiology and function, may help further understanding of endometrial proliferative disorders and guide future treatment strategies.

In vivo and in vitro assays were already developed to isolate and characterise endometrial stem cells [137,138,139]; a novel approach is to scrutinise endometrial stem cells with biospectroscopy.

Theophilou et al. [131] examined endometrial glands, using synchrotron- and focal- plane detector array-based FtIR spectroscopy. Vibrations of Amide I and II and PO_2_ in DNA and RNA nucleic acid were the main factors that allowed the segregation between the glands’ functionalis and basalis regions and the identification of putative stem cells within the deepest/terminal portion of the endometrial glands in the basalis layer. Furthermore, the putative stem cells were identified in two separate locations of the gland bases, prompting speculation that they might have different functions: one set being dormant and one in active differentiation [131].

The endometrial architecture was also explored by Patel et al. [94], who analysed benign and malignant uterine tissues with Raman microspectroscopy and were able to identify features of myometrium, glandular components and uterine epithelium. Again, different computer algorithms were tested to analyse the spectra obtained. The wavenumbers responsible for achieving the highest contrast between cellular structures were: 1234 cm^−1^ (Amide III), 1390 cm^−1^ (CH_3_ bend), 1675 cm^−1^ (Amide I/lipid), 1275 cm^−1^ (Amide III), 918 cm^−1^ (proline) and 936 cm^−1^ (proline, valine and proteins) [96]. By translating the spectral information obtained into spectra-derived contrast images, they were able to reconstruct an image of the tissue structure and match it with the correspondent Haematoxylin and Eosin (H&E) stained architecture.

The use of spectroscopy may, therefore, help to uncover structural and biological functions at a cellular level, leading a variety of potential clinical applications. Indeed, the identification of specific cell types, such as endometrial stem cells, and the observation of their biochemical activity, may correlate with clinical behaviours, such as chemotherapy resistance or tendency to metastasise, thus potentially informing the development of target treatments. The characterisation of the endometrial tissue structure, matched with corresponding histological appearance, may be used as a complementary and objective tool in support of standard histopathological diagnosis, with the aim of reducing inter-observer variability and human errors.

#### 4.3.2. Endometrial Cancer Diagnosis

The ability of FtIR spectroscopy to differentiate benign from malignant endometrial tissue, and to discriminate between different cancer subtypes, has been investigated in the quest for novel approaches to endometrial cancer diagnosis.

In a proof of concept study, Kelly et al. [132] used synchrotron-based Fourier-transform infrared microspectroscopy to examine de-waxed paraffin-embedded blocks of excised uterine tissue. They were able to discriminate between different endometrial cancer subtypes and benign tissues, as well as to distinguish between tamoxifen- and non-tamoxifen-associated cases. Interestingly, the cancer subtypes explored in the study exhibited different discriminating spectral patterns, which may reflect differences in biological characteristics: endometrioid types manifested discriminating wavenumbers, particularly in the protein region (1800–1480 cm^−1^), whereas those contributing the most to serous papillary or malignant mixed Mullerian tumours segregation were primarily in the DNA/RNA region (1425–900 cm^−1^) of the vibrational spectrum [132].

While the use of de-paraffinisation procedures allows access to large tissue banks, essential for retrospective and novel exploratory studies, these methods require tissue preparation or electronic spectral manipulation in order to exclude the contribution of paraffin to the tissue spectra [140].

An alternative method, proposed by Taylor et al. [133], is to immerse endometrial samples in ethanol-based liquid fixative (SurePath^TM^) and then perform sequential distilled H_2_O washes prior to spectral collection. In their study, instead of a synchrotron source, endometrial tissue was examined using a blackbody source in an ATR-FtIR instrument. The different phases of the menstrual cycle, could be clearly differentiated, exhibiting large differences in absorption bands in the lipid, Amide I, Amide II and asymmetric phosphate-stretching vibration regions. The authors also evaluated differences between benign and malignant endometrium. Multivariate analysis with principal component analysis (PCA), followed by linear discriminant analysis (LDA), obtained ~80% separation between benign and malignant spectra [133]. Among discriminating wavenumbers, most differences between benign and malignant tissues were consistent with previous data [132] and identified in the Amide I/II (1624, 1750, 1516 cm^−1^) regions, as well as in the protein band (1477 cm^−1^), asymmetrical PO_2_^−^ (1230 cm^−1^), RNA/carbohydrate (1168 cm^−1^) and phosphorylated proteins (968 cm^−1^). In addition, the lipid region (1735 cm^−1^) also appeared to have a significant discriminating role.

More recently, Depciuch et al. [9] identified the chemical changes that occur during the carcinogenesis process in endometrial tissues with both Raman and FtIR spectroscopy and reported classification accuracy ranging between 62.71 and 96.61%; the research group further combined Raman and FtIR spectroscopy to differentiate endometrial cancer, atypical hyperplasia and controls [10]. Alterations in nucleic acid, Amide I, lipids and collagen were seen in the Raman spectra, while changes in carbohydrates and amide vibrations were shown in the FtIR spectra. The shifts in wavenumber reported for tissues at different stages of carcinogenesis were consistent with those previously reported by other authors [94,133,141].

Table 2 and Table 3, illustrate a full list of typical FtIR absorption band assignment and Raman shifts, respectively, identified in endometrial cancers.

The ability of biospectroscopy to discriminate between normal and abnormal samples, rapidly providing label-free information on their biomolecular features bypassing the need for extensive tissue processing, has important clinical implications.

From a risk stratification point of view, biospectroscopy could be employed as a triage tool: the interrogation of endometrial biopsies at the bedside providing information on the likelihood of disease could help streamlining fast-track pathways. Prioritising conventional tissue diagnosis for those patients with the highest risk of cancer would not only speed up treatment, but also reduce patient anxiety that arises from waiting for test results.

Additionally, biospectroscopy could be developed into an intra-operative tool, to assess metastatic lymph node involvement or the nature of peritoneal deposits, to assist surgeons with precious real-time information, thus allowing them to individualise the treatment provided.

#### 4.3.3. Treatment and Surveillance

Beside discriminating normal and malignant tissues, optical spectroscopy techniques were explored for potential application in cancer therapy and disease surveillance. Multi-drug resistance is one of the greatest hurdles to chemotherapy and the ability to discriminate between sensitive/resistant cancer cell lines may improve treatment effectiveness and allow the development of individualised treatment plans.

Krishna et al. [134] reported on the identification by spectroscopy of specific phenotypes in uterine cancer cell lines. By applying PCA analysis to spectral data obtained with Raman and FtIR spectroscopes, multi-drug resistant (MDR) phenotypes of uterine sarcoma cell lines were distinguished from their drug sensitive counterparts, showing promising differentiation of cell types and the identification of their biological functions [134]. Discriminating spectral features included changes in protein secondary structure, DNA vibrations and lipid content.

Similarly, pilot studies have also examined pre- and post-chemotherapy tissue spectra of colon, breast and ovarian cancers, as follows.

Kaznowska et al. [145] applied PCA-LDA to FtIR spectra of cancerous and healthy colon tissues to detect pre- and post-chemotherapy spectral differences. Interestingly, although the baseline structure of healthy tissue was not restored after chemotherapy, the spectra of healthy and post-chemotherapy colon displayed a high degree of similarity, which could be used as a marker of treatment effectiveness: the higher the spectral similarities, the greater the treatment response [145]. Depciuch et al. [115] reported similar spectral findings in breast cancer tissue pre- and post-chemotherapy, where the resemblance of the post-chemotherapy tissue FtIR spectrum to the healthy tissue FtIR spectrum correlated with the clinical response to treatment.

Finally, Zendehdel et al. [146] studied chemotherapy resistance patterns of ovarian cancer cell lines with FtIR spectroscopy coupled with PCA analysis. The authors identified alterations in secondary protein structures and a shift toward the high wavenumbers of the CH_2_ stretching vibration to 2920 and 2852 cm^−1^.

The potential ability of spectroscopy methods to detect chemotherapy resistance and to assess spectral changes in tissues after treatment, could be exploited to guide the choice of cancer therapy and could be further developed in the context of disease monitoring.

### 4.4. Biospectroscopy of Biofluids: Screening and Cancer Diagnosis

With the endometrial cancer global disease burden expected to rise, disease screening, early detection and treatment monitoring will benefit from the development of more cost-effective, rapid, non-invasive and label-free techniques. Biological fluids, being readily accessible with low-cost procedures, represent the ideal sample target. Indeed, the study of biofluids with spectroscopy is becoming a rapidly emerging field and a number of pilot studies have now been published, focusing on oncological applications, as well as on a broad range of acute and chronic medical conditions [83,88,129,147,148,149,150,151,152,153].

With regards to endometrial cancer, biospectroscopy was proposed as a novel approach to test blood, urine and saliva (Table 4). The development of such techniques is particularly relevant as currently available methods, such as radiological imaging and blood biomarkers, lack the required sensitivity, specificity and accuracy to be used as effective screening tools. Interestingly, while Raman spectroscopy of blood serum was recently investigated for the first time as a non-invasive technique to diagnose endometriosis [154], no studies were found on the application of Raman spectroscopy to biofluids for EC diagnosis. 

Similar to experiments performed with endometrial tissue, the sample manipulations and chemometric analyses used to test biofluids vary between studies. The first pilot research, by Gajjar et al. [149], investigated the potential role of ATR-FtIR for cancer diagnosis using blood samples and, with the development of “machine classifiers”, reported classification rates of endometrial cancer versus controls up to 77.08% and 81.67% for serum and plasma, respectively. The same spectral data were more recently re-analysed by the research group [143], to evaluate the performance of alternative data-processing methods and classifier tools. Furthermore, the authors focused on the water-free sub-section of the spectrum (1430 cm^−1^ to 900 cm^−1^), in contrast with the more extended bio-fingerprint region (1800 cm^−1^ to 900 cm^−1^) used in the original paper. The researchers assessed four types of classifiers and reported high discrimination rates for both plasma (sensitivity of 0.865 ± 0.043 and specificity of 0.895 ± 0.023 with k-Nearest Neighbours algorithm) and serum (sensitivity 0.899 ± 0.023, specificity 0.763 ± 0.048 for LDA). This approach demonstrated for the first time the possibility of overcoming the dominant effect of water seen in the analysis of hydrated samples with MIR spectroscopy, which would support future applications of MIR spectroscopy in vivo to cancer diagnosis and screening [143].

Paraskevaidi et al. [155,156] used ATR-FtIR spectroscopy with PCA followed by support vector machine (PCA-SVM) to analyse blood plasma and serum from women with endometrial cancer and age-matched healthy controls. Test performance was described as high as 100% sensitivity and 85% specificity (98% accuracy) and changes in the bands associated with proteins and lipids were consistently responsible for the discrimination between blood plasma and serum from endometrial cancer and healthy samples. Traditionally, low-emissivity (low-E) slides have been used as ATR-FtIR substrates to support samples; however, their high cost may limit their use in large-scale studies and implementation in routine analysis. Aluminium foil substrate may represent an equivalent, cheaper, sample-support alternative for the detection of endometrial cancer with blood plasma and serum [156]. Indeed, Paraskevaidi et al. [156] showed that aluminium foil substrate was able to differentiate blood plasma and serum from patients with endometrial cancer and controls, with sensitivities and specificities comparable with the traditional low-E slides. The cost-effectiveness and ease of use, if validated in larger datasets, would greatly facilitate clinical application.

More recently, the same group, Paraskevaidi et al. [141], examined plasma from women with endometrial cancer, atypical hyperplasia and controls with ATR-FtIR in the largest diagnostic cross-sectional study to date (total n = 652). The study identified the six most discriminatory peaks for each subgroup analysis, suggesting these features could be developed in a panel of diagnostic spectral markers. Endometrial cancers and controls were differentiated with 87% sensitivity and 78% specificity (overall accuracy of 83%); further analysis of cancer subtypes achieved disease discrimination with sensitivities of 71–100% and specificities of 81–88%. In addition, the authors accounted for potential confounding factors, such as age, body mass index (BMI), diabetes, fasting status and blood pressure and found no impact on spectral classification after applying the MANOVA test (multivariate analysis of variance) to the spectral wavenumbers.

Discrimination between endometrial cancer and controls appears consistently superior for plasma over serum samples. It has been speculated that this might be due to the more complex and heterogeneous composition of the plasma; however, the cause of superior diagnostic results, being a panel of biomolecules or the presence of higher concentration of cell-free DNA, still remains to be determined [149,156].

In addition to plasma and serum, pilot research has also applied biospectroscopy techniques for endometrial cancer diagnosis to urine and saliva analysis.

Urine spectra obtained by Paraskevaidi et al. [152] yielded high levels of diagnostic accuracy after the application of multivariate analysis and classification algorithms (95% sensitivity and 100% specificity, 95% accuracy). The classification methods used included: partial least squares discriminant analysis (PLS-DA), PCA-SVM and genetic algorithm with LDA (GA-LDA.) The majority of discriminating wavelengths were once more located in the lipid, protein and acid nucleic infrared regions.

Saliva spectral analysis by Bel’skaya et al. [153] showed interesting alterations in the lipid regions of patients with endometrial and ovarian cancer; in particular, the ratio of the intensity of the absorption bands 2923/2957 cm^−1^ appeared to consistently decrease in cancer samples compared with controls, leading the authors to propose its use as a new diagnostic criterion.

Spectral differences in lipid absorption bands of healthy and diseased samples, also documented in prostate [157] and breast cancer [158], may be explained by tumour-mediated changes of the lipid metabolism and may warrant further investigations in the context of non-invasive endometrial cancer biomarker development.

## 5. State of Play, Present Challenges and Future Perspectives

We have reviewed here the progress made to date on the application of vibrational biospectroscopy methods to endometrial cancer studies and their role in the analysis of endometrial tissue and biofluids.

Vibrational biospectroscopy appears to be able to provide detailed information on endometrial architecture, and cell function. Furthermore, it was successfully used to discriminate between normal and cancerous tissues, to identify different cancer subtypes and to differentiate between drug-resistant and drug-sensitive cell phenotypes.

The qualitative and quantitative spectral changes associated with specific groups of biomolecules, such as proteins, lipids, amino acids and carbohydrates, can be exploited to develop disease-specific biomarkers.

The flexibility of the methods allows a variety of fresh, fixed, dry or wet specimens to be processed. An additional advantage is the ability of interrogating, simultaneously, the entirety of the molecules in a sample, in contrast with time-consuming individual biochemical mapping. With the development of computer software able to provide spectral data analysis and display in real time [159], there is an opportunity to conceptualise a novel bedside tool, or a point-of-care test for endometrial cancer. Such a tool, in conjunction with already established diagnostic tests, could facilitate patient triage and selection for additional more invasive procedures, and could be used intra-operatively to assess suspicious peritoneal lesions or metastatic lymph node involvement. Finally, the analysis of biofluids with spectroscopy might support the development of non-invasive screening pathways, as well as strategies for disease monitoring and assessment of response to treatment. Focus on the practical applications of biospectroscopy techniques to endometrial cancer diagnosis, screening and monitoring, therefore, fit nicely in the context of current endometrial cancer research priorities.

While vibrational spectroscopy development made significant progress over the last few decades, several challenges were, however, identified that still stand in the way of clinical translation; indeed, the techniques are still at the experimental stage and not yet in general use in clinics. More work is required, with adequately powered studies: from the adjustment of analytical instrumentation to be suitable for use in a clinical context, such a theatre room, a clinic or a laboratory, to the adaptation and standardisation of protocols for sample collection, processing and data analysis of different biological materials, as well as the essential need for spectral panel validation, reproducibility of results and design of analytical systems to fit well with clinical implementation [12].

These general considerations can also be extended to the application of biospectroscopy in Gynaecology. With regards to endometrial cancer, the first large study was published on the use of blood biospectroscopy as an early detection tool [141]; however, most of the other current evidence here-described consists of pilot and feasibility studies, with the inherent limitation of a small sample size. Patient characteristics, such as hormonal status, body mass index, age and co-morbidities, should be considered and patient groups appropriately matched to allow meaningful comparison between groups. Biofluids are ideal candidates for developing minimally invasive diagnostic/screening tests but, while preliminary studies have shown promising results with regards to the biospectroscopy diagnostic and screening potential for EC, current studies have not yet compared discrimination between EC and other cancers. The identification of disease-specific spectral features would be useful and should be explored prior to clinical implementation.

In addition, the majority of the experimental work in endometrial cancer has so far been conducted on preserved specimens, but to develop a point-of-care or intraoperative test, data are needed on the feasibility of performing reliable spectral analysis of fresh samples. In particular, the adverse impact on the quality of the data that may arise from the presence of water and blood components in the samples and the inherent heterogeneity of endometrial tissues are barriers that must be overcome in order to obtain results that are useful for clinical application. Furthermore, the variability of the sample-processing techniques used in different studies, even in the context of dry and fixed tissues, and the different approaches with regards to chemometric analysis and data extraction, make it challenging to draw direct comparison between datasets to date.

The limitations and challenges highlighted in this paper have important implications for future research. Validation and reproducibility of results should be assessed in larger scale, adequately powered, clinical trials. Future study designs should ensure patient groups are adequately matched. Spectral investigation of endometrial fresh tissue should be explored and results compared to dry analysis and “Gold Standard” histopathology. Finally, sample collection, processing and storage, as well as spectral acquisition and data extraction, should follow standardised protocols in order to minimise bias and guarantee the quality and reproducibility of results. Clearly, there is still a long way to go before clinical implementation, but vibrational biospectroscopy is proving to be an evolving technology with promising applications in endometrial cancer, which certainly warrants further exploration.

## Figures and Tables

**Table 1:** Spectroscopy of uterine tissues/cells, studies included in this Review.

Author	Year	Sample	No of Patients	Sample Preparation	Spectroscopy Method	Spectral Findings
Theophilou et al. [131]	2018	Benign uterine tissue	3 Multiple tissue sections per patient	Paraffin-fixed sections	Synchrotron FtIR (SR-FtIR) and globar focal plane array-based (FPA) FtIR	Identification of endometrial stem cell putative location with SR-FtIR: changes of stretching vibration in DNA, RNA, nucleic acids and protein secondary structure
						Differentiation between functionalis and basalis epithelial layers with FPA-FtIR: variation in protein secondary structure
Patel et al. [94]	2011	Benign and malignant endometrial tissues	4	Freshly-thawed frozen sections	Raman	Identification of tissue architecture: - high content of DNA and RNA in glandular epithelium - high protein content in collagenous stroma and myometrium
Kelly et al. [132]	2009	Benign and malignant endometrial tissues	26 Non-tamoxifen associated n = 15, tamoxifen-associated n = 8	Paraffin-fixed sections	Synchrotron FtIR (SR-FtIR)	Endometrioid carcinoma vs. benign: variations in protein content and secondary structure
						Serous papillary and malignant mixed mullerian tumours vs. benign: variations in RNA and DNA regions
						Group separation based on tamoxifen usage improved cancer vs. benign classification. Spectral changes were observed in protein secondary structure
Taylor et al. [133]	2011	Benign and malignant endometrial tissues	76	Ethanol-based fixed sections (SurePath^TM^)	ATR-FtIR	Stages of mentrual cycle classification: variation of lipid, Amide I, Amide II and asymmetric phosphate stretching vibration regions
						Cancer vs. benign: increased content of lipids and proteins
						Classification of tumour subtypes: - highest lipid content in grades I and III endometrioid, in clear cell tumours, adenosarcomas and carcinosarcomas - variation of protein secondary structure in endometrioid cancers
Depciuch et al. [9]	2021	Benign, pre-cancerous and malignant endometrial tissues	16 patients, 59 serial tissue samples	Paraffin-fixed sections	Raman and ATR-FtIR	Raman - cancer vs. benign: higher content of lipids and proteins and decreased collagen vibrations
						ATR-FtIR - cancer vs. benign: higher protein content and variations in protein secondary structure
Barnas et al. [10]	2020	Benign, pre-cancerous and malignant endometrial tissues	45	Paraffin-fixed sections	Raman and ATR-FtIR	Raman: - atypical hyperplasia vs. benign: higher nucleic acids, shift in protein and lipid peaks - cancer vs. benign: shift of nucleic acids, protein and lipid peaks
						ATR-FtIR: - atypical hyperplasia vs. benign: changes in carbohydrates, collagen and protein peak - cancer vs. control: changes in carbohydrates, collagen and protein peaks
Krishna et al. [134]	2005	Chemo- sensitive and multidrug resistant uterine sarcoma cell lines	15 samples	Cell culture	Raman and FtIR	Raman - multidrug resistant phenotype vs. sensitive cell line: changes in protein secondary structure and DNA vibrations
						FtIR - multidrug resistant phenotype vs. sensitive cell line: changes in protein secondary structure and lipid content

Key: ATR = attenuated total reflectance; DNA = deoxyribonucleic acid; FPA = focal plane array; FtIR = Fourier transform infrared; RNA = ribonucleic acid; SR = synchrotron; SurePath^TM^ is owned by TriPath Imaging.

**Table 2:** Peak assignment of typical FtIR spectra absorption bands observed in endometrioid and non-endometrioid endometrial cancers [9,131,133,142,143].

Endometrioid Wavenumber/(cm^−1^)	Non-Endometrioid Wavenumber/(cm^−1^)	Peak Assignment
1735	1736	Ester carbonyl of lipids
1682, 1624	1624, 1601	Amide I group in peptide linkages of proteins
1570, 1516	1570, 1516	Amide II group in peptide linkages of proteins
1535		C-N stretching contribution to Amide II
1477, 1462, 1450	1477	CH_2_ group scissoring modes in proteins (collagen)
1373		C-O-O symmetric stretching of fatty acids, and amino acid side chains
1340	1340	CH_2_ wagging of proline in amino acids and collagen
1240		Amide III-N-H bending, C-N stretch, C-C stretch of proteins, DNA, phospholipids
1234, 1230	1231	Asymmetric PO_2_^−^ stretching in RNA and DNA
1169	1173, 1142	C-O-C and C-O-P stretching and ring vibrations, symmetric C-O stretching coupled to C-O-H bending of carbohydrates
1088	1061	Symmetric PO_2_^−^ stretching in RNA and DNA
1066		C-O stretching mode of C–OH groups of serine, threonine, and tyrosine of protein
1034	1003	Symmetric C-O-C/C-O stretching of Glycogen
964	968	Phosphorylated proteins

**Table 3:** Raman shift, with corresponding vibrations described in endometrioid adenocarcinoma [9,10,94,144].

Raman Shift/(cm^−1^)	Vibration Assignment
853, 821	Proline, hydroxyproline, tyrosine, PO_2_ stretching from nucleic acids
880, 876	C-C stretching from proline and hydroxyproline
1066, 935	Proline, valine, PO_2_ stretching from nucleic acids
1299	Phosphodiester groups in nucleic acids
1302	Amide III (collagen assignment)
1335	Adenine
1376, 1374	Tryptophan
1447	CH_2_ bending from lipids and proteins
1561	C-C, tryptophan (protein assignment)
1685, 1660	Amide I
1792, 1723	C-O stretching from lipids
2795, 2758	CH_3_ stretching from lipids
2873	CH_2_ stretching from lipids

**Table 4:** Spectroscopy of biofluids: endometrial cancer studies included in this Review.

Author	Year	Sample	No of Patients	Preparation	Spectroscopy Method	Spectral Findings
Gajjar et al. [149]	2013	Endometrial cancer and healthy blood plasma and serum	60	Dried samples	ATR-FTIR	Plasma cancer vs. healthy: changes of stretching vibration in glycogen, RNA, fatty acids, amino acids and lower levels of lipids
						Serum cancer vs. healthy: changes of stretching vibration in DNA, RNA and lower levels of lipids
Paraskevaidi et al. [155]	2017	Endometrial cancer and healthy blood plasma and serum	89	Dried samples	ATR-FTIR	Discrimination between cancer subtypes for both plasma and serum due to protein and lipid alterations
Paraskevaidi et al. [156]	2018	Endometrial cancer and healthy blood plasma and serum	85 for plasma, 75 for serum	Dried samples	ATR-FTIR	Aluminium foil: - plasma cancer vs. healthy: changes in protein secondary structure and lipids - serum cancer vs. healthy: changes in glycogen, phosphate, fatty acid and amino acids
						Low-E slides: - plasma cancer vs. healthy: changes in protein secondary structure and lipids - serum cancer vs. healthy: changes in protein secondary structure and nucleic acids
Paraskevaidi et al. [152]	2018	Endometrial cancer and healthy urine	20	Dried samples	ATR-FTIR	Cancer vs. healthy: increased proteins and nucleic acids, decreased lipid content and alterations in protein secondary structure
Bel’skaya et al. [153]	2019	Endometrial cancer and controls saliva	55	Lipid extraction with Folch solution	FTIR	Cancer vs. controls: decreased lipid content
Paraskevaidi et al. [141]	2020	Endometrial cancer, atypical hyperplasia, and healthy blood plasma	652	Dried samples	ATR-FTIR	Cancer vs. healthy: increased lipids and decreased content of carbohydrates and fatty acids
						Hyperplasia vs. healthy: higher nucleic acids, collagen and stretching vibration in DNA and RNA
						Type I vs. Type II cancers: changes in protein secondary structure
Mabwa et al. [143]	2021	Endometrial cancer and healthy blood plasma and serum	60	Dried samples	ATR-FTIR	- Plasma and Serum (bio-fingerprint region 1430 cm^−1^ to 900 cm^−1^)—cancer vs. healthy: changes of stretching vibration in DNA, RNA, changes in fatty acid, amino acid and protein content

## References

[1] Bray F., Ferlay J., Soerjomataram I., Siegel R.L., Torre L.A., Jemal A. (2018). Global cancer statistics 2018: GLOBOCAN estimates of incidence and mortality worldwide for 36 cancers in 185 countries. CA Cancer J. Clin..

[2] Zhang S., Gong T.-T., Liu F.-H., Jiang Y.-T., Sun H., Ma X.-X., Zhao Y.-H., Wu Q.-J. (2019). Global, Regional, and National Burden of Endometrial Cancer, 1990–2017: Results from the Global Burden of Disease Study, 2017. Front. Oncol..

[3] Crosbie E., Morrison J. (2014). The emerging epidemic of endometrial cancer: Time to take action. Cochrane Database Syst. Rev..

[4] Brüggmann D., Ouassou K., Klingelhöfer D., Bohlmann M.K., Jaque J., Groneberg D.A. (2020). Endometrial cancer: Mapping the global landscape of research. J. Transl. Med..

[5] James Lind Alliance. https://www.jla.nihr.ac.uk/priority-setting-partnerships/womb-cancer/top-10-priorities.htm.

[6] Wan Y.L., Beverley-Stevenson R., Carlisle D., Clarke S., Edmondson R.J., Glover S., Holland J., Hughes C., Kitchener H.C., Kitson S. (2016). Working together to shape the endometrial cancer research agenda: The top ten unanswered research questions. Gynecol. Oncol..

[7] Pahlow S., Weber K., Popp J., Wood B.R., Kochan K., Rüther A., Perez-Guaita D., Heraud P., Stone N., Dudgeon A. (2018). Application of Vibrational Spectroscopy and Imaging to Point-of-Care Medicine: A Review. Appl. Spectrosc..

[8] Kendall C., Isabelle M., Bazant-Hegemark F., Hutchings J., Orr L., Babrah J., Baker R., Stone N. (2009). Vibrational spectroscopy: A clinical tool for cancer diagnostics. Anal..

[9] Depciuch J., Barnaś E., Skręt-Magierło J., Skręt A., Kaznowska E., Łach K., Jakubczyk P., Cebulski J. (2021). Spectroscopic evaluation of carcinogenesis in endometrial cancer. Sci. Rep..

[10] Barnas E., Skret-Magierlo J., Skret A., Kaznowska E., Depciuch J., Szmuc K., Łach K., Krawczyk-Marć I., Cebulski J. (2020). Simultaneous FTIR and Raman Spectroscopy in Endometrial Atypical Hyperplasia and Cancer. Int. J. Mol. Sci..

[11] Auner G.W., Koya S.K., Huang C., Broadbent B., Trexler M., Auner Z., Elias A., Mehne K.C., Brusatori M.A. (2018). Applications of Raman spectroscopy in cancer diagnosis. Cancer Metastasis Rev..

[12] Baker M.J., Byrne H.J., Chalmers J., Gardner P., Goodacre R., Henderson A., Kazarian S.G., Martin F.L., Moger J., Stone N. (2018). Clinical applications of infrared and Raman spectroscopy: State of play and future challenges. Analyst.

[13] Butler H.J., Ashton L., Bird B., Cinque G., Curtis K., Dorney J., Esmonde-White K., Fullwood N.J., Gardner B., Martin-Hirsch P.L. (2016). Using Raman spectroscopy to characterize biological materials. Nat. Protoc..

[14] Cui S., Zhang S., Yue S. (2018). Raman Spectroscopy and Imaging for Cancer Diagnosis. J. Health Eng..

[15] Kong K., Kendall C., Stone N., Notingher I. (2015). Raman spectroscopy for medical diagnostics—From in-vitro biofluid assays to in-vivo cancer detection. Adv. Drug Deliv. Rev..

[16] Nijssen A., Koljenovicć S., Schut T.C.B., Caspers P.J., Puppels G.J. (2009). Towards oncological application of Raman spectroscopy. J. Biophotonics.

[17] Kazarian S.G., Chan K.L.A. (2013). ATR-FTIR spectroscopic imaging: Recent advances and applications to biological systems. Analyst.

[18] Santos F., Magalhaes S., Henriques M.C., Fardilha M., Nunes A. (2018). Spectroscopic Features of Cancer Cells: FTIR Spectroscopy as a Tool for Early Diagnosis. Curr. Metab..

[19] Siqueira L.F.S., Lima K.M.G. (2016). MIR-biospectroscopy coupled with chemometrics in cancer studies. Analyst.

[20] Purandare N.C., Trevisan J., I Patel I., Gajjar K., Mitchell A.L., Theophilou G., Valasoulis G., Martin M., von Bünau G., Kyrgiou M. (2013). Exploiting biospectroscopy as a novel screening tool for cervical cancer: Towards a framework to validate its accuracy in a routine clinical setting. Bioanalysis.

[21] National Cancer Institute Endometrial Cancer Treatment Physician Data Query (PDQ). http://www.cancer.gov/cancertopics/pdq/treatment/endometrial/healthprofessional.

[22] Cancer Research UK. https://www.cancerresearchuk.org/health-professional/cancer-statistics/statistics-by-cancer-type/uterine-cancer/incidence.

[23] Office for National Statistics Cancer Survival by Stage at Diagnosis for England (Experimental Statistics)—Office for National Statistics. https://www.ons.gov.uk/peoplepopulationandcommunity/healthandsocialcare/conditionsanddiseases/datasets/cancersurvivalratescancersurvivalinenglandadultsdiagnosed.

[24] Lortet-Tieulent J., Ferlay J., Bray F., Jemal A. (2017). International Patterns and Trends in Endometrial Cancer Incidence, 1978–2013. JNCI J. Natl. Cancer Inst..

[25] Crosbie E., Zwahlen M., Kitchener H.C., Egger M., Renehan A. (2010). Body Mass Index, Hormone Replacement Therapy, and Endometrial Cancer Risk: A Meta-Analysis. Cancer Epidemiol. Biomark. Prev..

[26] Hecht J.L., Mutter G.L. (2006). Molecular and Pathologic Aspects of Endometrial Carcinogenesis. J. Clin. Oncol..

[27] Bokhman J.V. (1983). Two pathogenetic types of endometrial carcinoma. Gynecol. Oncol..

[28] Sherman M.E. (2000). Theories of Endometrial Carcinogenesis: A Multidisciplinary Approach. Mod. Pathol..

[29] Amant F., Moerman P., Neven P., Timmerman D., Van Limbergen E., Vergote I. (2005). Endometrial cancer. Lancet.

[30] Daley-Brown D., Oprea-Ilies G.M., Lee R., Pattillo R., Gonzalez-Perez R.R. (2015). Molecular cues on obesity signals, tumor markers and endometrial cancer. Horm. Mol. Biol. Clin. Investig..

[31] Creasman W.T., Odicino F., Maisonneuve P., Quinn M.A., Beller U., Benedet J.L., Heintz A., Ngan H.Y.S., Pecorelli S. (2006). Carcinoma of the Corpus Uteri. FIGO 26th Annual Report on the Results of Treatment in Gynecological Cancer. Int. J. Gynecol. Obstet..

[32] Murali R., Soslow R., Weigelt B. (2014). Classification of endometrial carcinoma: More than two types. Lancet Oncol..

[33] Talhouk A., McAlpine J.N. (2016). New classification of endometrial cancers: The development and potential applications of genomic-based classification in research and clinical care. Gynecol. Oncol. Res. Pract..

[34] Kandoth C., Schultz N., Cherniack A.D., Akbani R., Liu Y., Shen H., Robertson A.G., Pashtan I., Shen R., Benz C.C. (2013). Integrated genomic characterization of endometrial carcinoma. Nature.

[35] Church D.N., Stelloo E., Nout R.A., Valtcheva N., Depreeuw J., Ter Haar N., Noske A., Amant F., Tomlinson I.P.M., Wild P.J. (2015). Prognostic Significance of POLE Proofreading Mutations in Endometrial Cancer. JNCI J. Natl. Cancer Inst..

[36] Concin N., Matias-Guiu X., Vergote I., Cibula D., Mirza M.R., Marnitz S., Ledermann J., Bosse T., Chargari C., Fagotti A. (2021). ESGO/ESTRO/ESP guidelines for the management of patients with endometrial carcinoma. Int. J. Gynecol. Cancer.

[37] Luomaranta A., Leminen A., Loukovaara M. (2015). Magnetic Resonance Imaging in the Assessment of High-Risk Features of Endometrial Carcinoma: A Meta-Analysis. Int. J. Gynecol. Cancer.

[38] Andreano A., Rechichi G., Rebora P., Sironi S., Valsecchi M.G., Galimberti S. (2014). MR diffusion imaging for preoperative staging of myometrial invasion in patients with endometrial cancer: A systematic review and meta-analysis. Eur. Radiol..

[39] Cignini P., Vitale S.G., Laganà A.S., Biondi A., La Rosa V.L., Cutillo G. (2017). Preoperative work-up for definition of lymph node risk involvement in early stage endometrial cancer: 5-year follow-up. Updat. Surg..

[40] Koplay M., Dogan N.U., Erdoğan H., Sivri M., Erol C., Nayman A., Karabagli P., Paksoy Y., Çelik C. (2014). Diagnostic efficacy of diffusion-weighted MRI for pre-operative assessment of myometrial and cervical invasion and pelvic lymph node metastasis in endometrial carcinoma. J. Med. Imaging Radiat. Oncol..

[41] Wouk N., Helton M. (2019). Abnormal Uterine Bleeding in Premenopausal Women. Am. Fam. Physician.

[42] Gill K.A. (2014). Chapter 1: Introduction to Diagnostic Ultrasound. Ultrasound in Obstetrics and Gynaecology.

[43] Cusimano M.C., Simpson A.N., Han A., Hayeems R., Bernardini M.Q., Robertson D., Kives S.L., Satkunaratnam A., Baxter N.N., Ferguson S.E. (2019). Barriers to care for women with low-grade endometrial cancer and morbid obesity: A qualitative study. BMJ Open.

[44] Bosch T.V.D. (2012). Ultrasound in the diagnosis of endometrial and intracavitary pathology: An update. Australas. J. Ultrasound Med..

[45] Clarke M.A., Long B.J., Del Mar Morillo A., Arbyn M., Bakkum-Gamez J.N., Wentzensen N. (2018). Association of Endometrial Cancer Risk With Postmenopausal Bleeding in Women: A Systematic Review and Meta-analysis. JAMA Intern. Med..

[46] Timmermans A., Opmeer B.C., Khan K.S., Bachmann L.M., Epstein E., Clark T.J., Gupta J.K., Bakour S.H., Bosch T.V.D., van Doorn H.C. (2010). Endometrial Thickness Measurement for Detecting Endometrial Cancer in Women With Postmenopausal Bleeding. Obstet. Gynecol..

[47] Smith-Bindman R., Kerlikowske K., Feldstein V.A., Subak L.L., Scheidler J., Segal M.R., Brand R., Grady D. (1998). Endovaginal Ultrasound to Exclude Endometrial Cancer and Other Endometrial Abnormalities. JAMA.

[48] Getpook C., Wattanakumtornkul S. (2006). Endometrial thickness screening in premenopausal women with abnormal uterine bleeding. J. Obstet. Gynaecol. Res..

[49] Nicula R., Costin N. (2015). Management of endometrial modifications in perimenopausal women. Clujul Med..

[50] Bignardi T., Van den Bosch T., Condous G. (2009). Abnormal uterine and post-menopausal bleeding in the acute gynaecology unit. Best Pract. Res. Clin. Obstet. Gynaecol..

[51] Valle R.F. (2008). Operative Hysteroscopy. Glob. Libr. Women’s Med..

[52] British Gynaecological Cancer Society (2017). BGCS Uterine Cancer Guidelines: Recommendations for Practice.

[53] Bakour S.H., Jones S.E., O’Donovan P. (2006). Ambulatory hysteroscopy: Evidence-based guide to diagnosis and therapy. Best Pract. Res. Clin. Obstet. Gynaecol..

[54] Kremer C., Barik S., Duffy S. (1998). Flexible outpatient hysteroscopy without anaesthesia: A safe, successful and well tolerated procedure. Br. J. Obstet. Gynaecol..

[55] Clark T.J., Voit D., Gupta J.K., Hyde C., Song F., Khan K.S. (2002). Accuracy of Hysteroscopy in the Diagnosis of Endometrial Cancer and Hyperplasia. JAMA.

[56] Royal College of Obstetricians and Gynaecologists Diagnostic Hysteroscopy under General Anaesthesia. Consent Advice 1..

[57] Relph S., Lawton T., Broadbent M., Karoshi M. (2016). Failed hysteroscopy and further management strategies. Obstet. Gynaecol..

[58] Farquhar C., Ekeroma A., Furness S., Arroll B. (2003). A systematic review of transvaginal ultrasonography, sonohysterography and hysteroscopy for the investigation of abnormal uterine bleeding in premenopausal women. Acta Obstet. Gynecol. Scand..

[59] Van Dongen H., De Kroon C.D., Jacobi C.E., Trimbos J.B., Jansen F.W. (2007). Diagnostic hysteroscopy in abnormal uterine bleeding: A systematic review and meta-analysis. BJOG Int. J. Obstet. Gynaecol..

[60] National Institute for Health and Care Excellence Heavy Menstrual Bleeding: Assessment and Management. NICE Guideline [NG88]..

[61] National Institute for Health and Care Excellence Heavy Menstrual Bleeding (Update). A: Evidence Reviews for Diagnostic Test Accuracy in Investigation for Women Presenting with Heavy Menstrual Bleeding. https://www.nice.org.uk/guidance/ng88/evidence/a-diagnostic-test-accuracy-pdf-4782293101.

[62] Slaoui M., Fiette L. (2011). Histopathology procedures: From tissue sampling to histopathological evaluation. Methods Mol. Biol..

[63] Morelli P., Porazzi E., Ruspini M., Restelli U., Banfi G. (2013). Analysis of errors in histology by root cause analysis: A pilot study. J. Prev. Med. Hyg..

[64] Phillips V. (2005). Results of a questionnaire regarding criteria for adequacy of endometrial biopsies. J. Clin. Pathol..

[65] Breijer M.C., Visser N.C.M., van Hanegem N., van der Wurff A.A., Opmeer B.C., van Doorn H.C., Mol B.W.J., Pijnenborg J.M.A., Timmermans A. (2016). A Structured Assessment to Decrease the Amount of Inconclusive Endometrial Biopsies in Women with Postmenopausal Bleeding. Int. J. Surg. Oncol..

[66] van Hanegem N., Prins M.M., Bongers M.Y., Opmeer B.C., Sahota D., Mol B.W.J., Timmermans A. (2016). The accuracy of endometrial sampling in women with postmenopausal bleeding: A systematic review and meta-analysis. Eur. J. Obstet. Gynecol. Reprod. Biol..

[67] Narice B.F., Delaney B., Dickson J.M. (2018). Endometrial sampling in low-risk patients with abnormal uterine bleeding: A systematic review and meta-synthesis. BMC Fam. Pract..

[68] Gordon S., Westgate J. (1999). The incidence and management of failed Pipelle sampling in a general outpatient clinic. Aust. N. Z. J. Obstet. Gynaecol..

[69] Visser N.C., Breijer M.C., Herman M.C., Bekkers R.L., Veersema S., Opmeer B.C., Mol B.W., Timmermans A., Pijnenborg J.M. (2013). Factors attributing to the failure of endometrial sampling in women with postmenopausal bleeding. Acta Obstet. Gynecol. Scand..

[70] Williams A.R.W., Brechin S., Porter A.J.L., Warner P., Critchley H.O.D. (2008). Factors affecting adequacy of Pipelle and Tao Brush endometrial sampling. BJOG Int. J. Obstet. Gynaecol..

[71] Van Doorn H.C., Opmeer B.C., Burger C.W., Duk M.J., Kooi G.S., Mol B.W.J. (2007). Dutch Study in Postmenopausal Bleeding (DUPOMEB) Inadequate office endometrial sample requires further evaluation in women with postmenopausal bleeding and abnormal ultrasound results. Int. J. Gynecol. Obstet..

[72] Mohanlal R.D. (2020). Endometrial sampling at an academic hospital in South Africa: Histological findings, lessons learnt and interesting surprises. Afr. J. Lab. Med..

[73] Du J., Li Y., Lv S., Wang Q., Sun C., Dong X., He M., Ulain Q., Yuan Y., Tuo X. (2016). Endometrial sampling devices for early diagnosis of endometrial lesions. J. Cancer Res. Clin. Oncol..

[74] Clark T.J., Mann C.H., Shah N., Khan K.S., Song F., Gupta J.K. (2002). Accuracy of outpatient endometrial biopsy in the diagnosis of endometrial cancer: A systematic quantitative review. BJOG: Int. J. Obstet. Gynaecol..

[75] Dijkhuizen F.P., Mol B.W., Brölmann H.A., Heintz A.P. (2000). The accuracy of endometrial sampling in the diagnosis of patients with endometrial carcinoma and hyperplasia: A meta-analysis. Cancer.

[76] Feldman S., Berkowitz R.S., Tosteson A.N. (1993). Cost-effectiveness of strategies to evaluate postmenopausal bleeding. Obstet. Gynecol..

[77] Clark T.J., Mann C.H., Shah N., Khan K.S., Song F., Gupta J.K. (2001). Accuracy of outpatient endometrial biopsy in the diagnosis of endometrial hyperplasia. Acta Obstet. Gynecol. Scand..

[78] World Health Organisation Screening. https://www.who.int/cancer/prevention/diagnosis-screening/screening/en/.

[79] Møller P., Seppälä T., Bernstein I., Holinski-Feder E., Sala P., Evans D.G., Lindblom A., Macrae F., Blanco I., Sijmons R. (2017). Cancer incidence and survival in Lynch syndrome patients receiving colonoscopic and gynaecological surveillance: First report from the prospective Lynch syndrome database. Gut.

[80] Jacobs I., Gentry-Maharaj A., Burnell M., Manchanda R., Singh N., Sharma A., Ryan A., Seif M.W., Amso N., Turner G. (2010). Sensitivity of transvaginal ultrasound screening for endometrial cancer in postmenopausal women: A case-control study within the UKCTOCS cohort. Lancet Oncol..

[81] Clark T., Barton P., Coomarasamy A., Gupta J., Khan K. (2006). Gynaecological oncology: Investigating postmenopausal bleeding for endometrial cancer: Cost-effectiveness of initial diagnostic strategies. BJOG Int. J. Obstet. Gynaecol..

[82] Jacobs I.J., Menon U., Ryan A., Gentry-Maharaj A., Burnell M., Kalsi J.K., Amso N., Apostolidou S., Benjamin E., Cruickshank D. (2016). Ovarian cancer screening and mortality in the UK Collaborative Trial of Ovarian Cancer Screening (UKCTOCS): A randomised controlled trial. Lancet.

[83] Baker M.J., Hussain S.R., Lovergne L., Untereiner V., Hughes C., Lukaszewski R.A., Thiéfin G., Sockalingum G.D. (2016). Developing and understanding biofluid vibrational spectroscopy: A critical review. Chem. Soc. Rev..

[84] Byrne H.J., Baranska M., Puppels G.J., Stone N., Wood B., Gough K.M., Lasch P., Heraud P., Sulé-Suso J., Sockalingum G.D. (2015). Spectropathology for the next generation: Quo vadis?. Analyst.

[85] Seddon A.B., Napier B., Lindsay I., Lamrini S., Moselund P.M., Stone N., Bang O., Farries M. (2018). Prospective on using fibre mid-infrared supercontinuum laser sources for in vivo spectral discrimination of disease. Analyst.

[86] Mantsch H.H., Moss D. (2011). Foreword. Biomedical Applications of Synchrotron Infrared Microspectroscopy.

[87] Baker M.J., Trevisan J., Bassan P., Bhargava R., Butler H.J., Dorling K.M., Fielden P.R., Fogarty S.W., Fullwood N.J., Heys K.A. (2014). Using Fourier transform IR spectroscopy to analyze biological materials. Nat. Protoc..

[88] Mitchell A.L., Gajjar K.B., Theophilou G., Martin F.L., Martin-Hirsch P.L. (2014). Vibrational spectroscopy of biofluids for disease screening or diagnosis: Translation from the laboratory to a clinical setting. J. Biophotonics.

[89] The International Organisation for Standardisation ISO 20473: 2007 Optics and Photonics. Spectral Bands 2007, 10. www.iso.org/standard/39482.html.

[90] Stuart B. (2004). Infrared Spectroscopy: Fundamentals and Applications.

[91] Filik J., Frogley M.D., Pijanka J.K., Wehbe K., Cinque G. (2012). Electric field standing wave artefacts in FTIR micro-spectroscopy of biological materials. Analyst.

[92] Bassan P., Lee J., Sachdeva A., Pissardini J., Dorling K.M., Fletcher J.S., Henderson A., Gardner P. (2013). The inherent problem of transflection-mode infrared spectroscopic microscopy and the ramifications for biomedical single point and imaging applications. Analyst.

[93] Raman C.V., Krishnan K.S. (1928). A New Type of Secondary Radiation. Nature.

[94] Patel I.I., Trevisan J., Evans G., Llabjani V., Martin-Hirsch P.L., Stringfellow H.F., Martin F.L. (2011). High contrast images of uterine tissue derived using Raman microspectroscopy with the empty modelling approach of multivariate curve resolution-alternating least squares. Analyst.

[95] Kirsch M., Schackert G., Salzer R., Krafft C. (2010). Raman spectroscopic imaging for in vivo detection of cerebral brain metastases. Anal. Bioanal. Chem..

[96] Cui L., Butler H.J., Martin-Hirsch P.L., Martin F.L. (2016). Aluminium foil as a potential substrate for ATR-FTIR, transflection FTIR or Raman spectrochemical analysis of biological specimens. Anal. Methods.

[97] Crow P., Stone N., Kendall C.A., Uff J.S., Farmer J.A.M., Barr H., Wright M.P.J. (2003). The use of Raman spectroscopy to identify and grade prostatic adenocarcinoma in vitro. Br. J. Cancer.

[98] Theophilou G., Lima K.M.G., Briggs M., Martin-Hirsch P.L., Stringfellow H.F., Martin F.L. (2015). A biospectroscopic analysis of human prostate tissue obtained from different time periods points to a trans-generational alteration in spectral phenotype. Sci. Rep..

[99] Patel I.I., Trevisan J., Singh P.B., Nicholson C.M., Krishnan R.K.G., Matanhelia S.S., Martin F.L. (2011). Segregation of human prostate tissues classified high-risk (UK) versus low-risk (India) for adenocarcinoma using Fourier-transform infrared or Raman microspectroscopy coupled with discriminant analysis. Anal. Bioanal. Chem..

[100] Güler G., Guven U., Oktem G. (2019). Characterization of CD133^+^/CD44^+^ human prostate cancer stem cells with ATR-FTIR spectroscopy. Analyst.

[101] German M., Hammiche A., Ragavan N., Tobin M., Cooper L.J., Matanhelia S.S., Hindley A.C., Nicholson C.M., Fullwood N.J., Pollock H.M. (2006). Infrared Spectroscopy with Multivariate Analysis Potentially Facilitates the Segregation of Different Types of Prostate Cell. Biophys. J..

[102] Bergholt M.S., Zheng W., Lin K., Ho K.Y., Teh M., Yeoh K.G., So J., Huang Z. (2011). In Vivo Diagnosis of Esophageal Cancer Using Image-Guided Raman Endoscopy and Biomolecular Modeling. Technol. Cancer Res. Treat..

[103] Yao H.-W., Liu Y.-Q., Fu W., Shi X.-Y., Zhang Y.-F., Xu Y.-Z. (2011). Initial research on Fourier transform infrared spectroscopy for the diagnosis of colon neoplasms. Guang Pu Xue Yu Guang Pu Fen Xi Guang Pu.

[104] Sahu R.K., Argov S., Walfisch S., Bogomolny E., Moreh R., Mordechai S. (2010). Prediction potential of IR-micro spectroscopy for colon cancer relapse. Analyst.

[105] Nallala J., Piot O., Diebold M.-D., Gobinet C., Bouché O., Manfait M., Sockalingum G.D. (2014). Infrared and Raman Imaging for Characterizing Complex Biological Materials: A Comparative Morpho-Spectroscopic Study of Colon Tissue. Appl. Spectrosc..

[106] Maziak D.E., Do M.T., Shamji F.M., Sundaresan S.R., Perkins D.G., Wong P.T. (2007). Fourier-transform infrared spectroscopic study of characteristic molecular structure in cancer cells of esophagus: An exploratory study. Cancer Detect. Prev..

[107] Cameron J.M., Butler H.J., Smith B.R., Hegarty M.G., Jenkinson M.D., Syed K., Brennan P.M., Ashton K., Dawson T., Palmer D.S. (2019). Developing infrared spectroscopic detection for stratifying brain tumour patients: Glioblastoma multiforme vs. lymphoma. Analyst.

[108] Bury D., Morais C.L.M., Martin F.L., Lima K.M.G., Ashton K.M., Baker M.J., Dawson T.P. (2020). Discrimination of fresh frozen non-tumour and tumour brain tissue using spectrochemical analyses and a classification model. Br. J. Neurosurg..

[109] Kong K., Zaabar F., Rakha E., Ellis I., Koloydenko A., Notingher I. (2014). Towards intra-operative diagnosis of tumours during breast conserving surgery by selective-sampling Raman micro-spectroscopy. Phys. Med. Biol..

[110] Zúñiga W.C., Jones V., Anderson S.M., Echevarria A., Miller N.L., Stashko C., Schmolze D., Cha P.D., Kothari R., Fong Y. (2019). Raman Spectroscopy for Rapid Evaluation of Surgical Margins during Breast Cancer Lumpectomy. Sci. Rep..

[111] Nargis H., Nawaz H., Ditta A., Mahmood T., Majeed M., Rashid N., Muddassar M., Bhatti H., Saleem M., Jilani K. (2019). Raman spectroscopy of blood plasma samples from breast cancer patients at different stages. Spectrochim. Acta Part A Mol. Biomol. Spectrosc..

[112] Lyng F.M., Traynor D., Nguyen T.N.Q., Meade A., Rakib F., Al-Saady R., Goormaghtigh E., Al-Saad K., Ali M.H. (2019). Discrimination of breast cancer from benign tumours using Raman spectroscopy. PLoS ONE.

[113] Walsh M.J., Holton S.E., Kajdacsy-Balla A., Bhargava R. (2012). Attenuated total reflectance Fourier-transform infrared spectroscopic imaging for breast histopathology. Vib. Spectrosc..

[114] Tian P., Zhang W., Zhao H., Lei Y., Cui L., Wang W., Li Q., Zhu Q., Zhang Y., Xu Z. (2015). Intraoperative diagnosis of benign and malignant breast tissues by fourier transform infrared spectroscopy and support vector machine classification. Int. J. Clin. Exp. Med..

[115] Depciuch J., Kaznowska E., Zawlik I., Wojnarowska-Nowak R., Cholewa M., Heraud P., Cebulski J. (2016). Application of Raman Spectroscopy and Infrared Spectroscopy in the Identification of Breast Cancer. Appl. Spectrosc..

[116] Depciuch J., Kaznowska E., Golowski S., Koziorowska A., Zawlik I., Cholewa M., Szmuc K., Cebulski J. (2017). Monitoring breast cancer treatment using a Fourier transform infrared spectroscopy-based computational model. J. Pharm. Biomed. Anal..

[117] Huang Z., McWilliams A., Lui H., McLean D.I., Lam S., Zeng H. (2003). Near-infrared Raman spectroscopy for optical diagnosis of lung cancer. Int. J. Cancer.

[118] Sun X., Xu Y., Wu J., Zhang Y., Sun K. (2013). Detection of lung cancer tissue by attenuated total reflection–Fourier transform infrared spectroscopy—A pilot study of 60 samples. J. Surg. Res..

[119] Lui H., Zhao J., McLean D., Zeng H. (2012). Real-time Raman Spectroscopy for In Vivo Skin Cancer Diagnosis. Cancer Res..

[120] Lima C.A., Goulart V.P., Côrrea L., Pereira T.M., Zezell D.M. (2015). ATR-FTIR Spectroscopy for the Assessment of Biochemical Changes in Skin Due to Cutaneous Squamous Cell Carcinoma. Int. J. Mol. Sci..

[121] Lima C.A., Goulart V.P., Correa L., Zezell D.M. (2016). Using Fourier transform infrared spectroscopy to evaluate biological effects induced by photodynamic therapy. Lasers Surg. Med..

[122] Walsh M.J., Singh M.N., Pollock H.M., Cooper L.J., German M.J., Stringfellow H.F., Fullwood N.J., Paraskevaidis E., Martin-Hirsch P.L., Martin F.L. (2007). ATR microspectroscopy with multivariate analysis segregates grades of exfoliative cervical cytology. Biochem. Biophys. Res. Commun..

[123] Kelly J.G., Angelov P.P., Trevisan J., Vlachopoulou A., Paraskevaidis E., Martin-Hirsch P.L., Martin F.L. (2010). Robust classification of low-grade cervical cytology following analysis with ATR-FTIR spectroscopy and subsequent application of self-learning classifier eClass. Anal. Bioanal. Chem..

[124] Kelly J.G., Cheung K.T., Martin C., O’Leary J.J., Prendiville W., Martin-Hirsch P.L., Martin F.L. (2010). A spectral phenotype of oncogenic human papillomavirus-infected exfoliative cervical cytology distinguishes women based on age. Clin. Chim. Acta.

[125] Halliwell D.E., Morais C.L., Lima K.M., Trevisan J., Siggel-King M.R., Craig T., Ingham J., Martin D.S., Heys K., Kyrgiou M. (2017). An imaging dataset of cervical cells using scanning near-field optical microscopy coupled to an infrared free electron laser. Sci. Data.

[126] Ramos I.R., Meade A.D., Ibrahim O., Byrne H.J., McMenamin M., McKenna M., Malkin A., Lyng F.M. (2016). Raman spectroscopy for cytopathology of exfoliated cervical cells. Faraday Discuss..

[127] Krishna C.M., Prathima N.B., Malini R., Vadhiraja B.M., Bhatt R.A., Fernandes D.J., Kushtagi P., Vidyasagar M.S., Kartha V.B. (2006). Raman spectroscopy studies for diagnosis of cancers in human uterine cervix. Vib. Spectrosc..

[128] Paraskevaidi M., Ashton K.M., Stringfellow H.F., Wood N.J., Keating P.J., Rowbottom A.W., Martin-Hirsch P.L., Martin F.L. (2018). Raman spectroscopic techniques to detect ovarian cancer biomarkers in blood plasma. Talanta.

[129] Lima K.M.G., Gajjar K.B., Martin-Hirsch P.L., Martin F.L. (2015). Segregation of ovarian cancer stage exploiting spectral biomarkers derived from blood plasma or serum analysis: ATR-FTIR spectroscopy coupled with variable selection methods. Biotechnol. Prog..

[130] Frost J., Ludeman L., Hillaby K., Gornall R., Lloyd G., Kendall C., Shore A.C., Stone N. (2016). Raman spectroscopy and multivariate analysis for the non invasive diagnosis of clinically inconclusive vulval lichen sclerosus. Analyst.

[131] Theophilou G., Morais C.L.M., Halliwell D.E., Lima K.M.G., Drury J., Martin-Hirsch P.L., Stringfellow H.F., Hapangama D.K., Martin F.L. (2018). Synchrotron- and focal plane array-based Fourier-transform infrared spectroscopy differentiates the basalis and functionalis epithelial endometrial regions and identifies putative stem cell regions of human endometrial glands. Anal. Bioanal. Chem..

[132] Kelly J.G., Singh M., Stringfellow H.F., Walsh M.J., Nicholson J.M., Bahrami F., Ashton K.M., Pitt M.A., Martin-Hirsch P.L., Martin F.L. (2009). Derivation of a subtype-specific biochemical signature of endometrial carcinoma using synchrotron-based Fourier-transform infrared microspectroscopy. Cancer Lett..

[133] Taylor S.E., Cheung K.T., Patel I.I., Trevisan J., Stringfellow H.F., Ashton K.M., Wood N.J., Keating P.J., Martin-Hirsch P.L., Martin F.L. (2011). Infrared spectroscopy with multivariate analysis to interrogate endometrial tissue: A novel and objective diagnostic approach. Br. J. Cancer.

[134] Murali Krishna C., Kegelaer G., Adt I., Rubin S., Kartha V., Manfait M., Sockalingum G. (2005). Characterisation of uterine sarcoma cell lines exhibiting MDR phenotype by vibrational spectroscopy. Biochim. Biophys. Acta (BBA)—Gen. Subj..

[135] Figueira P.G.M., Abrão M.S., Krikun G., Taylor H.S. (2011). Stem cells in endometrium and their role in the pathogenesis of endometriosis. Ann. N. Y. Acad. Sci..

[136] Rosen J.M., Jordan C.T. (2009). The Increasing Complexity of the Cancer Stem Cell Paradigm. Science.

[137] Schwab K.E., Gargett C.E. (2007). Co-expression of two perivascular cell markers isolates mesenchymal stem-like cells from human endometrium. Hum. Reprod..

[138] Wolff E.F., Wolff A.B., Du H., Taylor H.S. (2007). Demonstration of Multipotent Stem Cells in the Adult Human Endometrium by In Vitro Chondrogenesis. Reprod. Sci..

[139] Gargett C.E., Chan R., Schwab K.E. (2007). Endometrial stem cells. Curr. Opin. Obstet. Gynecol..

[140] Nallala J., Lloyd G.R., Stone N. (2015). Evaluation of different tissue de-paraffinization procedures for infrared spectral imaging. Analyst.

[141] Paraskevaidi M., Morais C.L.M., Ashton K.M., Stringfellow H.F., McVey R.J., Ryan N.A.J., O’Flynn H., Sivalingam V.N., Kitson S.J., Mackintosh M.L. (2020). Detecting Endometrial Cancer by Blood Spectroscopy: A Diagnostic Cross-Sectional Study. Cancers.

[142] Krishna C.M., Kegelaer G., Adt I., Rubin S., Kartha V.B., Manfait M., Sockalingum G.D. (2006). Combined Fourier transform infrared and Raman spectroscopic approach for identification of multidrug resistance phenotype in cancer cell lines. Biopolymers.

[143] Mabwa D., Gajjar K., Furniss D., Schiemer R., Crane R., Fallaize C., Martin-Hirsch P.L., Martin F.L., Kypraios T., Seddon A.B. (2021). Mid-infrared spectral classification of endometrial cancer compared to benign controls in serum or plasma samples. Analyst.

[144] Movasaghi Z., Rehman S., Rehman I.U. (2007). Raman Spectroscopy of Biological Tissues. Appl. Spectrosc. Rev..

[145] Kaznowska E., Depciuch J., Szmuc K., Cebulski J. (2017). Use of FTIR spectroscopy and PCA-LDC analysis to identify cancerous lesions within the human colon. J. Pharm. Biomed. Anal..

[146] Zendehdel R., Masoudi-Nejad A., Mohammadzadeh J., Shirazi F.H. (2012). Cisplatin Resistant Patterns in Ovarian Cell Line Using FTIR and Principle Component Analysis. Iran J. Pharm. Res..

[147] Owens G., Gajjar K., Trevisan J., Fogarty S.W., Taylor S.E., Da Gama-Rose B., Martin-Hirsch P.L., Martin F.L. (2014). Vibrational biospectroscopy coupled with multivariate analysis extracts potentially diagnostic features in blood plasma/serum of ovarian cancer patients. J. Biophotonics.

[148] Taleb I., Thiéfin G., Gobinet C., Untereiner V., Bernard-Chabert B., Heurgué A., Truntzer C., Hillon P., Manfait M., Ducoroy P. (2013). Diagnosis of hepatocellular carcinoma in cirrhotic patients: A proof-of-concept study using serum micro-Raman spectroscopy. Analyst.

[149] Gajjar K., Trevisan J., Owens G., Keating P.J., Wood N.J., Stringfellow H.F., Martin-Hirsch P.L., Martin F.L. (2013). Fourier-transform infrared spectroscopy coupled with a classification machine for the analysis of blood plasma or serum: A novel diagnostic approach for ovarian cancer. Analyst.

[150] Hands J.R., Clemens G., Stables R., Ashton K., Brodbelt A., Davis C., Dawson T.P., Jenkinson M.D., Lea R.W., Walker C. (2016). Brain tumour differentiation: Rapid stratified serum diagnostics via attenuated total reflection Fourier-transform infrared spectroscopy. J. Neuro-Oncol..

[151] Yu M.-C., Rich P., Foreman L., Smith J., Tanna A., Dibbur V., Unwin R., Tam F.W.K. (2017). Label Free Detection of Sensitive Mid-Infrared Biomarkers of Glomerulonephritis in Urine Using Fourier Transform Infrared Spectroscopy. Sci. Rep..

[152] Paraskevaidi M., Morais C.L.M., Lima K.M.G., Ashton K.M., Stringfellow H.F., Martin-Hirsch P.L., Martin F.L. (2018). Potential of mid-infrared spectroscopy as a non-invasive diagnostic test in urine for endometrial or ovarian cancer. Analyst.

[153] Bel’Skaya L.V., Sarf E.A., Solomatin D.V., Kosenok V.K. (2019). Analysis of the lipid profile of saliva in ovarian and endometrial cancer by IR fourier spectroscopy. Vib. Spectrosc..

[154] Parlatan U., Inanc M.T., Ozgor B.Y., Oral E., Bastu E., Unlu M.B., Basar G. (2019). Raman spectroscopy as a non-invasive diagnostic technique for endometriosis. Sci. Rep..

[155] Paraskevaidi M., Morais C., Raglan O., Lima K.M.G., Martin-Hirsch P.L., Paraskevaidis E., Kyrgiou M., Martin F.L. (2017). Spectroscopy of blood samples for the diagnosis of endometrial cancer and classification of its different subtypes. J. Clin. Oncol..

[156] Paraskevaidi M., Morais C.L.M., Raglan O., Lima K.M.G., Paraskevaidis E., Martin-Hirsch P.L., Kyrgiou M., Martin F.L. (2018). Aluminium foil as an alternative substrate for the spectroscopic interrogation of endometrial cancer. J. Biophotonics.

[157] Baker M.J., Gazi E., Brown M.D., Shanks J.H., Gardner P., Clarke N.W. (2008). FTIR-based spectroscopic analysis in the identification of clinically aggressive prostate cancer. Br. J. Cancer.

[158] Fabian H., Thi N.A.N., Eiden M., Lasch P., Schmitt J., Naumann D. (2006). Diagnosing benign and malignant lesions in breast tissue sections by using IR-microspectroscopy. Biochim. Biophys. Acta (BBA)—Biomembr..

[159] Trevisan J., Angelov P., Scott A.D., Carmichael P.L., Martin F.L. (2013). IRootLab: A free and open-source MATLAB toolbox for vibrational biospectroscopy data analysis. Bioinformatics.

